# EU FP7 research funding for an orphan drug (Orfadin®) and vaccine (Hep C) development: a success and a failure?

**DOI:** 10.1186/s40545-021-00317-8

**Published:** 2021-04-28

**Authors:** L. Schmidt, O. Sehic, C. Wild

**Affiliations:** HTA Austria- Austrian Institute for Health Technology Assessment GmbH, Garnisongasse 7/20, 1090 Vienna, Austria

**Keywords:** Research funding, Public funding, Return on investment, Late-stage clinical development

## Abstract

**Background:**

We considered the extent of the contribution of publicly funded research to the late-stage clinical development of pharmaceuticals and medicinal products, based on the European Commission (EC) FP7 research funding programme. Using two EC FP7-HEALTH case study examples—representing two types of outcomes—we then estimated wider public and charitable research funding contributions.

**Methods:**

Using the publicly available database of FP7-HEALTH funded projects, we identified awards relating to late-stage clinical development according to the systematic application of inclusion and exclusion criteria, classified them according to product type and clinical indication, and calculated total EC funding amounts. We then identified two case studies representing extreme outcomes: failure to proceed with the product (hepatitis C vaccine) and successful market authorisation (Orfadin® for alkaptonuria). Total public and philanthropic research funding contributions to these products were then estimated using publicly available information on funding.

**Results:**

12.3% (120/977) of all EC FP7-HEALTH awards related to the funding of late-stage clinical research, totalling € 686,871,399. Pharmaceutical products and vaccines together accounted for 84% of these late-stage clinical development research awards and 70% of its funding. The hepatitis C vaccine received total European Community (FP7 and its predecessor, EC Framework VI) funding of €13,183,813; total public and charitable research funding for this product development was estimated at € 77,060,102. The industry sponsor does not consider further development of this product viable; this now represents public risk investment. FP7 funding for the late-stage development of Orfadin® for alkaptonuria was so important that the trials it funded formed the basis for market authorisation, but it is not clear whether the price of the treatment (over €20,000 per patient per year) adequately reflects the substantial public funding contribution.

**Conclusions:**

Public and charitable research funding plays an essential role, not just in early stage basic research, but also in the late-stage clinical development of products prior to market authorisation. In addition, it provides risk capital for failed products. Within this context, we consider further discussions about a public return on investment and its reflection in pricing policies and decisions justified.

## Background

The recent COVID-19 pandemic shows how joint research ventures between public institutions and industry, supported by large amounts of public funding, are able to generate fast and effective responses to the scientific challenges we face. In this paper, we seek to add to the body of work that recognises the contribution that public funding makes to the development of successful pharmaceuticals and medical products and the implications that the public return on public investment should have for subsequent pricing policies and decisions. Following on from our earlier work [1], we seek to draw attention to the fact that public funding also plays an important role in the development of products that subsequently prove unsuccessful, thus demonstrating the risk capital that public funding provides, mirroring the risk capital invested by the pharmaceutical industry. Risk capital refers to funds invested in ventures with some positive prospects but no guarantee of success. These funds are risked without any guarantee of return, in exchange for the possibility of generating high rewards.

There is an ongoing debate about the relative role of public funding of biomedical research and the contribution of the pharmaceutical industry to drug discovery and development. Some see the central scientific contributions to drug development, including basic and applied science, as coming from industry [2]. However, a considerable body of research now exists to testify to the fact that public and philanthropic funding of research and development (R&D) activities is pivotal to the development and approval of pharmaceuticals and medicinal products, although this does not appear to be reflected in the pricing policies of such products. In addition to the direct funding of R&D activities, there are tax deductions and tax credits for drug development, particularly in the case of orphan drugs, which can significantly lower manufacturer costs [3]. An analysis reported by Global Justice Now estimates that the public pays for two-thirds of all upfront R&D drug costs and that around one-third of all medicines originate in research institutions in the public sector [4]. Similarly, a more recent study looking at 248 Food and Drug Administration (FDA) drug approvals over a 10-year period found that 19% originated in publicly supported research and development and a further 6% originated in companies spun out from publicly supported research programmes [5]. This mirrors earlier work, which reported that 24% of new drugs approved by the FDA had their origins in university transfers to pharmaceutical or biotechnology companies [6] and that of 15 clinically important drugs, public sector research made key enabling discoveries for 11 of them [7].

In recent years, a focus of the pharmaceutical industry has been on developing orphan drugs; in 2015 65% of new active substances first approved by the FDA, the European Medicines Agency (EMA) and Health Canada were specialty drugs [3], often for rare diseases. These new treatments for rare diseases are often very expensive. One estimate puts the median annual price for each patient per year treated with top selling orphan drugs in the United States (US) in 2016 at $83 883, which represents 5.5 times the median annual cost for leading non-orphan drugs [3]. Prices for orphan drugs are higher than for non-orphan drugs, despite the fact that the clinical costs of drug development for orphan drugs are estimated to be approximately half that of a non-orphan drug [8]. Approvals for specialised drugs, particularly orphan drugs, are often based on smaller trials (reflecting the size of the disease populations) with consequently lower development costs [3].

It was our intention to investigate the EU-research funding contributions to pharmaceutical and medicinal product development and to contribute to the body of knowledge on public investments in drug R&D. Our starting point for looking at public funding was the 7th EU Framework Programme for Research and Technology (FP7, 2007–2013), which was one of the largest research and technological development programmes in the world. It accounts for the third largest share of the European Union (EU) budget and was the main financial instrument to build the European Research Area [9]. We were interested in identifying public European Union funding for the late-stage clinical development of products. After summarising the number and value of awards that related to the late-stage clinical development of pharmaceuticals and medicinal products funded by this programme, we look in more detail at two FP7 case studies. One case study is an example of a rare disease drug for which public funding was pivotal in securing marketing approval. The other case study is a vaccine candidate for which development has stalled, which is an example of extensive public risk capital.

## Methods

We applied a multistep process by first identifying European Community funding of late-stage clinical research and then second, by identifying two case studies that illustrated extreme scenarios for public funding.

An analysis of the publicly available excel spreadsheet [10], showing EC funding of research projects as part of the EC FP7 programme, enabled us to identify the number of awards relating to the late-stage clinical development of pharmaceuticals or medical devices, and classify them according to disease area and type of product. Inclusion/exclusion criteria: we included biomedical research projects relating to clinical development and excluded awards relating to basic research. We included awards which mentioned any of the following keywords in the title or project summary: “first-in-human”, “proof of concept”, “clinical trial”, “trial”, “clinical evaluation”, “effectiveness” or “safety”. We then classified each included project according to the type of medical device or other health care technology being researched (pharmaceutical, medicine product/device, vaccine) and the stated disease area for product application.

Following the analysis of the EC FP7 data, we then identified two case studies from the dataset, where the publicly funded R&D related to specific products with large-scale industry involvement, and where we could demonstrate two extreme outcome scenarios relating to public investment for illustrative purposes. One was an example of public funding for a specialty drug that was pivotal in securing successful market authorisation as an orphan drug (nitisinone for alkaptonuria) and one as an example of significant public risk capital where there was no market authorisation (hepatitis C vaccine). We estimated the public and philanthropic contribution to both.

In estimating the wider public and philanthropic contribution to the development of both products, we employed a search strategy similar to that previously used and described in more detail elsewhere [1]. However, this time we also decided to include funding of research work cited in the references of patent applications. As Nayak [5] has suggested, defining the research that justifies patent claims on the drug is one way to assess the contributions of various parties and sectors in the drug development continuum. We therefore searched: patent documents, for research named as part of the development process; clinicaltrials.gov to identify any relevant clinical trials; the NIH Reporter database to identify funding of key researchers named in the patents; references of FP7 project reports for relevant research; US Securities and Exchange Commission filings (EDGAR); and references of review articles documenting the development of the two products [11–15].

To identify financial contributions to the development of nitisinone originating from public and philanthropic sources, we additionally looked for publications listed in scientific documents regarding market authorisation of Orfadin® in Australia (AUSPAR [16]), USA (FDA [17]) and Europe (EMA: EPAR [18]). For Orfadin®, we were also able to identify a report from the Canadian Agency for Drugs and Technologies in Health (CADTH) regarding the use of nitisinone for hereditary tyrosinemia type 1 (HT-1) [19] and a health technology briefing report from the NIHR Innovation Observatory regarding its use in alkaptonuria [20]. Documents on market authorisation or reimbursement were not available for the hepatitis C case study given its failure to proceed to market authorisation application.

## Results

### EC FP7 Cordis programme (2007–2013)

25,388 projects received funding as part of the EC FP7 programme, of which 977 were funding awards in the health area (coded as FP7-HEALTH programme awards). 120 of these 977 FP7 HEALTH Cordis awards, equating to 12.3% of the total, were identified as relating to the funding of late-stage clinical research of new pharmaceutical products, gene therapies or devices, prior to marketing authorisation. Total EC expenditure on the 120 awards was documented as € 686,871,399.41 and, with the exception of one award, all funding awards included large industry or SME partners.

Table 1 shows the type of product that was the subject of the funding award (Table 1) and the disease or medical specialty area that the identified product related to (Table 2). As can be seen from Table 1, 55% (66/120) of products receiving FP7 funding were pharmaceutical products, accounting for almost €375 million.

**Table 1:** Type of products receiving FP7 grants

Type of product	Number of FP7 product-related awards	Total EC funding across awards
Pharmaceutical product	66	€ 374,566,636.44
Medicine product/device	26	€ 132,960,884.58
Vaccine	18	€ 103,555,184.34
Gene therapy/regenerative therapy/cell therapy	10	€ 75,788,694.05
Total	120	€ 686,871,399.41

**Table 2:** Disease/medical specialty area to which the product relates

Disease/specialty area	Number of FP7 product-related awards	Total EC funding across awards
Neonatal/paediatric	27	€ 144,697,453.51
Infection	22	€ 103,803,065.19
Cancer	19	€ 98,467,149.13
Cardiovacular	7	€ 53,059,136.35
Diabetes	7	€ 39,387,415.80
Neurology	5	€ 39,361,768.70
Rheumatology	5	€ 38,211,526.50
Neurodegenerative	4	€ 26,594,086.00
Immunology	4	€ 25,825,968.00
Genetic diseases	4	€ 22,702,610.55
Eye/ear	4	€ 20,227,976.98
Respiratory	3	€ 17,974,237.00
Orthopaedics	2	€ 17,649,160,30
Regenerative medicine	1	€ 11,935,340.00
Psychiatry	1	€ 5,994,380.00
Skin	1	€ 4,961,654,00
Gastroenterology	1	€ 4,951,792.40
Brain	1	€ 4,865,656,00
Endocrinology	1	€ 3,308,154.00
Urology	1	€ 2,892,869.00
Total	120	€ 686,871,399.41

Table 2 shows that just over one-fifth (*n* = 27/120, 22.5%) of products related to neonatal or paediatric care. Products relating to infections (*n* = 22/120, 18.3%) and cancer (*n* = 19/120, 15.8%) were also well represented. These three indications accounted for around 50% of total EC funding contributions for late-stage clinical development research.

### Case study 1: vaccine for hepatitis C

Our first case study of an FP7-funded product is an example of public risk investment. Several reviews testify to the difficulties in developing a vaccine for hepatitis C, often citing the lack of appropriate animal models for preclinical vaccine development, the enormous genetic diversity of chronic hepatitis C virus (HCV) and limited at-risk populations for testing vaccine efficacy [11, 21]. Despite these difficulties, the clinical-stage biopharmaceutical company Okairos AG had been developing a hepatitis C vaccine based on its novel virus vectors technology platform. The AdCh3NSmut1/MVA-NSmut vaccine is intended to prevent HCV infection in adults. In 2013 GlaxoSmithKline (GSK) acquired Okairos to enable the further expansion of its vaccines platform technology [22]. However, in 2019 the National Institute of Allergy and Infectious Diseases (NIAID) reported that a phase I/II clinical trial of Okairos’ AdCh3NSmut1/MVA-NSmut vaccine had failed to demonstrate effectiveness in protecting against chronic HCV infection, when compared to placebo [23]. The GSK Annual Report from 2019 (Vaccines chapter) subsequently states [24]:

“To focus our work, we also terminated our hepatitis C virus and universal flu programmes as they had not met our expectations.”

Considerable public and philanthropic funds had been invested into the development of this vaccine, representing non-industry risk capital investment. Development of the AdCh3NSmut1/MVA-NSmut vaccine for hepatitis C was carried out within the EC FP7 PEACHI project (number 305632), which received an EU contribution of € 4,388,813. The preceding HEPACIVAC project was funded within the EC Framework VI and received an EU contribution of € 8,795,000. In addition, there was a grant (BMH4982239) as part of the EC FP4 research programme between 1998 and 2001, although the exact amount could not be determined. We identified 15 separate NIH grants for research surrounding this vaccine development. Other national-level grants were from Germany (two grants) and the UK (four awards). We found mention of Japanese and Korean government grants, but these were not traceable.

In terms of philanthropic and charitable funding, we were able to document grants and financial support from the American Cancer Society, Susan G. Comer Breast Cancer Foundation, Juvenile Diabetes Research Foundation and Cystic Fibrosis Foundation. The breadth of charitable organisations supporting the researchers working on the hepatitis C vaccine shows how basic research is often applicable to several indications, especially at an early stage of development.

Total funding from public and philanthropic sources in the development of this vaccine to date that we could identify amounted to € 13,183,813 from EU sources, GBP 3,075,667 from the UK and US $ 69,146,795 from U.S. sources, which converted to a common Euro currency, resulted in a minimum total value of € 77,060,102 (using reference exchange rates, last 12 months^1^). Importantly, GSK retaining patent rights to the vaccine development, despite not wishing to proceed itself, would prevent others in the public or private sectors taking up development.

### Case study 2: orphan drug Orfadin® (nitisinone)

Next, we looked at an example of a product where public European funding was instrumental in clinical development, and which subsequently led to successful market authorisation. Orfadin® (nitisinone) is currently approved for the treatment of hereditary tyrosinemia type 1 and in 2020 it received a positive opinion from the Committee for Medicinal Products for Human Use of EMA for the treatment of adult patients with alkaptonuria (AKU) [25]. It was originally developed for clinical use by two Swedish scientists working at the University of Gothenburg in 1989 and was subsequently commercialised by Swedish Orphan Biovitrum International AB (Sobi), receiving approval by the FDA in 2002 and the EMA in 2005 for use in the treatment of HT-1. The following clinical trials of nitisinone for AKU were identified: the phase two study named SONIA 1 (NCT01828463), the phase three study named SONIA 2 (NCT01916382) and the phase two study NCT00107783 (long-term study of nitisinone to treat alkaptonuria). We also looked at public funding of the previous studies known as “NTBC” [26] and “Quebec” [27], that had assessed the efficacy and safety of nitisinone for the original indication of HT-1.

Approval of nitisinone for use among AKU patients was based on the results of the DevelopAKUre programme (project number: 304985). The DevelopAKUre programme of clinical development with an overall FP7 programme budget of around €11.3 million received an EC contribution of just under €6 million. There were a number of public (university and research organisations) and philanthropic (patient organisations) partners in the consortium, in addition to the four private for-profit entities (including Sobi), in receipt of EU funds, who would likely have made their own financial (through overheads) or in-kind contributions to the project. The DevelopAKUre programme funded the SONIA 1, SONIA 2 and SOFIA clinical trials.

In addition, we identified general developmental work for nitisinone, which had drawn upon two NIH grants totalling just over $1 million and around GBP 700,000 from the Childwick Trust and AKU Society. We were able to document public funding (though not specific amounts) of earlier research into the development of nitisinone from: National Human Genome Research Institute; FDA; USA Department of Energy; NIH Child Health and Human Development; National Institute of Diabetes and Digestive and Kidney Diseases; Swedish Medical Research Council, the Deutsche Forschungsgemeinschaft (DFG); the University of Leipzig and the University of Montreal. In addition, philanthropic funding (but not specific amounts) was identified from the charitable Swedish Cancer Society, Swedish Cancer Foundation, the Children’s Cancer Foundation of Sweden, Inga Britt and Arne Lundbergs Foundation, the March of Dimes Foundation, the Groupe d'Aide aux Enfants Tyrosinémiques du Québec and the Garrod Society of Canada. The UK National AKU Centre, funded by NHS England, also supported.

The Cordis project 304985 final report summary for DevelopAKUre states:

“According to the EU orphan drug legislation of 2000, nitisinone will be granted market exclusivity for AKU within the EU for 10 years if marketing authorisation is successful. This would provide a significant incentive for Sobi to distribute nitisinone to AKU patients in a sustainable manner…. Sobi will exploit this license to generate revenue for the company, helping create jobs and brand value….. Treatment will be long-term, thus providing financial incentives for Sobi as the Marketing Authorisation Holder”

We question whether nitisinone is being distributed in a sustainable manner, given its price. According to the EPAR positive opinion, the recommended dose in the adult AKU population is 10 mg once daily [28]. This equates to an annual cost of just over GBP 17,000, according to estimated drug cost data [20]. The per patient price of this treatment for alkaptonuria in Europe is around € 20,671, based on social insurance prices in Austria [29]. A pharmacoeconomic review [30] (conducted in conjunction with the CADTH review into Orfadin® for the treatment of HT-1) found the use of nitisinone in combination with dietary restriction of tyrosine and phenylalanine not likely to be cost-effective at a willingness-to-pay threshold of Canadian $ 200,000 per QALY.

## Discussion

In some discussions, it is implied that late-stage clinical research is funded entirely by private pharmaceutical companies [31]. However, we have shown here that around 12% of EC FP7 HEALTH funding was used to fund late-stage clinical research, including funding for clinical trials used for drug approval. We have shown that the European public funding programme contributed considerably to the funding of the late-stage clinical development of products. We add to the body of evidence showing that publicly supported research plays a major role in the late-stage development of drugs, as recently demonstrated by Nayek et al. [5].

Horizon 2020 (FP8) covers the years 2014–2020 with a budget of 80 billion euros, of which 7.9 billion euros was invested in biomedical and health research projects. The largest percentage of projects was in the areas of biotechnological research and development activities (28.28%) and basic research (26.95%); these two sectors together accounted for 46.99% of the health budget [32]. Health Europe (FP9) will cover the period 2021–2027. Each funding cycle undergoes extensive evaluation in terms of the scope of the framework, the percentage of projects submitted and approved, the funding instruments applied and the perceived impacts of the programme [33].

The Orfadin® case study shows the central importance of public funding of clinical trials, upon which market authorisation may wholly rest. Although the public funding amounts for Orfadin® were relatively modest in comparison with the huge sum for the hepatitis C vaccine, the trials receiving public funds supported by the DevelopAKUre programme (largely financed by FP7 funding) represent the main body of trials evidence on which marketing authorisation was granted. Hence the pivotal and almost exclusive contribution of public funding to this orphan drug indication can clearly be seen. The Orfadin® example confirms previous conclusions that despite pharmaceuticals often arising from late-stage contributions from publicly funded research, intellectual property rights underlying these drugs are transferred to private sector manufacturers [34]. There is no mention of a fair pricing mechanism or a return on investment of the public investment in the DevelopAKUre project report.

An argument often given for the pricing of pharmaceuticals is the considerable risk capital that the pharmaceutical industry is required to deploy; risk capital that is often unrewarded, as in the case of unsuccessful product development [35]. Whilst this is undoubtedly true, we would like to draw attention to the similarly large degree of risk capital that the public sector may deploy in the search for new products, as the case of the hepatitis C vaccine has shown here.

Limitations of our analysis include the fact that we omitted an analysis of public expenditure in the form of tax subsidies. We are unable to comment on the relative importance and weighting of public and industry funding, unlike one report into the tuberculosis drug bedaquiline, which put total public expenditures at 3.1–5.1 times those of the originator company, or 1.6–2.2 when the cost of failures and foregone investment opportunities are counted [36]. Furthermore, public contributions to clinical studies may take the form of resources that represent in-kind contributions; again not captured here. Lastly, we only used funding information as specified by the authors themselves in their publications. Even where authors specified funding information, it was not always possible to trace the exact funding amounts as there are no standards requiring funders to report details of grants and awards.

We therefore support the argument that publicly sponsored research plays an important role in late-stage product development, a fact that should inform policies related to drug pricing and the fair return on public sector investment [5]. We follow earlier authors in hoping that our analysis can contribute to the debate on a fair return on investment of public investment. We agree that an accurate and transparent determination of all the costs going into drug development—and from which sectors—is required to inform ongoing discussions on how to best foster develop and reasonably pay for innovation [37]. Transparency regarding public and philanthropic funding of pharmaceuticals and medicinal products is needed to ensure that the public return on investment can be adequately reflected in price building, which itself is in need of transparency.

## Conclusion

In this paper, we have shown the considerable extent to which the Cordis EC FP7 programme of public financing contributed to the late-stage clinical research of products destined for commercial marketing; funding worth a total of almost € 690 million. We selected two case studies representing product development that had received FP7 funding to show two extremes of funding outcomes. The first was the unsuccessful development of a vaccine for the hepatitis C virus, which—at just over € 77 million (of which €13 million came from FP7 or its predecessor funding programme)—shows the extent to which public and philanthropic funding represent huge amounts of risk investment. The second case study related to the clinical development of Orfadin® for alkaptonuria, which went on to receive market authorisation, the clinical results for which were entirely based on the publicly funded EC FP7 DevelopAKUre project.

This study adds to the body of evidence demonstrating that public funding of medical research is relevant to the late-stage clinical development of products. Furthermore, the FP7 funding programme is not the only source of EC funding for medicines. The European Commission also contributes to the Innovative Medicines Initiative, which is made up of funding from EU tax payers and the pharmaceutical industry, although we could find no evidence of IMI funding in the two case studies examined. We contribute to the debate on the pricing of pharmaceuticals by demonstrating the importance of public funding both for market authorisation and in terms of the risk capital that public funds provide.

## Data Availability

Available on request from corresponding author.

## Abbreviations

AKU: Alkaptonuria
AUSPAR: Australian Public Assessment Report for Prescription Medicines
CADTH: Canadian Agency for Drugs and Technologies in Health
DFG: Deutsche Forschungsgemeinschaft
EC: European Commission
EDGAR: Electronic Data Gathering, Analysis and Retrieval
EMA: European Medicines Agency
EU: European Union
EPAR: European Public Assessment Report
FDA: Food and Drug Administration
FP7: Framework Programme for Research and Technology
GSK: GlaxoSmithKline
HT-1: Hereditary tyrosinemia type 1
NHS: National Health Service
NIH: National Institutes of Health
R&D: Research and development
Sobi: Swedish Orphan Biovitrum AB
SME: Small to Medium Enterprise
UK: United Kingdom
US: United States

## Footnotes

1 https://www.ecb.europa.eu/stats/policy_and_exchange_rates/euro_reference_exchange_rates/html/eurofxref-graph-gbp.en.html; https://www.ecb.europa.eu/stats/policy_and_exchange_rates/euro_reference_exchange_rates/html/eurofxref-graph-usd.en.html
